# Association Between Social Distancing Compliance and Public Place Crowding During the COVID-19 Pandemic: Cross-Sectional Observational Study Using Computer Vision to Analyze Surveillance Footage

**DOI:** 10.2196/50929

**Published:** 2025-04-17

**Authors:** Lasse Suonperä Liebst, Wim Bernasco, Peter Ejbye-Ernst, Nigel van Herwijnen, Thomas van der Veen, Dennis Koelma, Cees G M Snoek, Marie Rosenkrantz Lindegaard

**Affiliations:** ^1^Department of Sociology, University of Copenhagen, Copenhagen, Denmark; 2 Netherlands Institute for the Study of Crime and Law Enforcement, Amsterdam, The Netherlands; 3 Department of Human Geography, Planning and International Development Studies, University of Amsterdam, Amsterdam, The Netherlands; 4 Department of Social and Cultural Anthropology, Universitat Autònoma de Barcelona, Barcelona, Spain; 5 Department of Social and Political Sciences, Università degli Studi di Milano, Milan, Italy; 6 Informatics Institute, Faculty of Science, University of Amsterdam, Amsterdam, The Netherlands; 7 Department of Sociology, Faculty of Social and Behavioural Sciences, University of Amsterdam, Amsterdam, The Netherlands

**Keywords:** social distancing, compliance, crowding, urban public spaces, computer vision, surveillance footage, COVID-19 pandemic

## Abstract

**Background:**

Social distancing behavior has been a critical nonpharmaceutical measure for mitigating the COVID-19 pandemic. For this reason, there has been widespread interest in the factors determining social distancing violations, with a particular focus on individual-based factors.

**Objective:**

In this paper, we examine an alternative and less appreciated indicator of social distancing violations: the situational opportunity for maintaining interpersonal distance in crowded settings. This focus on situational opportunities is borrowed from criminology, where it offers an alternative to individual-based explanations of crime and rule violations. We extend this approach to the COVID-19 pandemic context, suggesting its relevance in understanding distancing compliance behavior.

**Methods:**

Our data comprise a large collection of video clips (n=56,429) from public places in Amsterdam, the Netherlands, captured by municipal surveillance cameras throughout the first year of the pandemic. We automatized the analysis of this footage using a computer vision algorithm designed for pedestrian detection and estimation of metric distances between individuals in the video still frames. This method allowed us to record social distancing violations of over half a million individuals (n=539,127) across more and less crowded street contexts.

**Results:**

The data revealed a clear positive association between crowding and social distancing violations, evident both at the individual level and when aggregated per still frame. At the individual level, the analysis estimated that each additional 10 people present increased the likelihood of a distancing violation by 9 percentage points for a given pedestrian. At the aggregated level, there was an estimated increase of approximately 6 additional violations for every 10 additional individuals present, with a very large *R*² of 0.80. Additionally, a comparison with simulation data indicated that street spaces should, in principle, provide sufficient room for people to pass each other while maintaining a 1.5-meter distance. This suggests that pedestrians tend to gravitate toward others, even when ample space exists to maintain distance.

**Conclusions:**

The direct positive relationship between crowding and distancing violations suggests that potential transmission encounters can be identified by simply counting the number of people present in a location. Our findings thus provide a reliable and scalable proxy measure of distancing noncompliance that offers epidemiologists a tool to easily incorporate real-life behavior into predictive models of airborne contagious diseases. Furthermore, our results highlight the need for scholars and public health agencies to consider the situational factors influencing social distancing violations, especially those related to crowding in public settings.

## Introduction

On the recommendation of the world’s leading public health organizations, most countries around the globe installed and enforced social distancing measures to avoid close stranger contact and crowded settings during the COVID-19 pandemic. This is in line with evidence showing that social distancing is an effective strategy to limit the number of cases and deaths of COVID-19 [1]. However, the mitigating effect of these measures obviously depends on public compliance, which might be limited since people often do not adhere to health recommendations [2]. Consequently, public health agencies and researchers have closely monitored adherence to COVID-19 measures throughout the pandemic, tracking both compliance levels and influencing factors.

A primary research instrument for these evaluations has been repeated surveys [3]. This research approach has identified a number of individual-level compliance predictors, including risk perceptions, normative attitudes, and age [4]. This line of research has also identified situational correlates of social distancing compliance, relating to one’s opportunity to violate [5]. For example, one survey-based study found that people who reported going outside for nonessential activities were more likely to violate social distancing and stay-at-home measures [6]. However, studies investigating such situational factors are relatively scarce, and therefore, called for in the literature [4]—plausibly reflecting the circumstance that self-reports are less suitable for measuring the situational dimension of people’s everyday lives and COVID-19 health routines [7,8] (though it has been successfully done [9]). More broadly, this mirrors the issue that self-reports are known to offer a coarse-grained picture of how people behave in here-and-now situations [10].

This study addresses this research gap by applying a methodological approach that is increasingly recognized as the gold standard for microdetailed examinations of human behavior in everyday situations: video observation [11,12]. This method also addresses a recent call for unobtrusive, naturalistic observations of health behaviors, a technique that has been underused in health research [13]. Specifically, we leveraged a unique dataset of more than 60,000 hours of video of public place behavior captured by security cameras throughout the first year of the COVID-19 pandemic. To analyze the video footage, we used a computer vision algorithm, which we have developed and reliability-tested [14]. Computer vision algorithms are at the frontier of video data analysis research but remain rare in empirical studies of social and health behaviors [13,15].

This analysis, with a sample size exceeding half a million individuals, represents the most comprehensive observational study of social distancing behavior in public settings to date. It is also the first to use an automated, and thus, truly scalable approach. Building on our previous smaller-scale studies using manually coded video data [16,17], we hypothesize a positive association between crowding and noncompliance with social distancing guidelines (note that the video data for these 2 cited studies were also sampled in Amsterdam during the pandemic, although we stress that there is no direct data overlap between the current and these prior studies) [16,17]. As such, this study may be considered a large-scale replication of the prior studies, addressing the call for more replication studies in the social-behavioral sciences [18]. In addition, we mention that the current data and overall findings are cited in a separate paper outlining the technical aspects of the computer vision algorithm [14]. Specifically, we expect that as the number of people in a given space increases, so does the likelihood of close encounters [19]. This is particularly true in highly crowded areas where maintaining the recommended interpersonal distance becomes physically challenging [20]. Our focus on crowding as a situational factor in explaining distancing violations marks a shift from the literature’s emphasis on individual-level predictors. This approach aligns with a “situational opportunity” perspective used in criminology to explain the occurrence of rule-breaking and crime [19]. This implies a focus on the opportunities people have to violate rules when engaging in their everyday routines and environments, irrespective of who they are.

In our previous 2 papers, we empirically established a positive correlation between crowd size and social distancing violations [16,17]. However, we did not examine the precise nature of this relationship in detail—whether the functional form of the slope is linear or follows a curved pattern. Moreover, a positive relationship between the number of people present and social distancing is neither obvious nor trivial, as observing the presence of others may change individuals’ behavior [21]. A classical illustration of this is research on bystander intervention in emergencies, which shows that the presence of additional bystanders may reduce each bystander’s willingness to intervene [22]. If this reduction is substantial, it could outweigh the safety-in-numbers dynamic where a larger crowd offers more opportunities for helping victims (though see Philpot et al [23]). Consequently, an increase in the number of bystanders may counterintuitively reduce the likelihood of any bystander intervening. Similarly, increasing the number of people in a place might not only make it physically more challenging to maintain distance from others but also promote greater vigilance and make individuals more focused on keeping their distance.

## Methods

### Study Design

Data were video footage of public behavior captured by 49 municipal surveillance cameras located in relatively busy outdoor public areas of Amsterdam, the Netherlands. Data access was provided by the Amsterdam police with permission from the Netherlands Public Prosecution Service (PaG/BJZ/49986).

The footage was recorded on Thursdays and Saturdays between 9 AM and 8 PM during the first year of the COVID-19 pandemic, from the beginning of March 2020 to June 2021. Throughout this period, the Dutch Government recommended that citizens kept a 1.5-meter distance in public spaces. Constrained by limited police resources to convert, store, and transport video recordings, we chose Thursdays and Saturdays to ensure that both the weekday and weekend behavior was included in the sample. The computer vision algorithm used for the automatic data coding was developed from the convolutional neural network model for pedestrian detection by Hasan et al [24], and in turn, extended with a linear regression model to estimate the metric distance between pedestrians.

We used the algorithm to analyze still frames, in total 56,429, and selected each full hour for all cameras across the available footage. For each frame, we recorded the total number of people present (ie, the people crowding predictor). Note that we excluded all still frames with fewer than 2 persons, given that we are interested in how the presence of other people may influence the likelihood of social distancing violations—that is, if alone, one cannot be involved in any close encounters. Since we did not use facial recognition or other identification software, we do not know if the same individuals reappeared in the footage at different times and on different cameras. For each of the 539,127 persons observed, the computer vision algorithm further estimated the number of other persons within a 1.5-meter radius, which is a social distancing violation as defined in the Netherlands (ie, the outcome: 0=no social distancing violation; 1=at least 1 violation).

It should be emphasized that in the vast majority of situations, crowding levels did not make it practically impossible to avoid social distancing violations. On average, the viewsheds of the cameras captured around 650 m² of walkable street surface. While an area of this size could theoretically contain up to 433 individuals without 1.5-meter distancing violations, the median number of individuals observed in a still frame was 7 (SD 7.7), which would give each individual an average personal space of no less than 93 m^2^. At this low level of crowding, individuals are not forced to violate social distancing rules for lack of physical space, even if some parts of the space-covered area are not available for pedestrians. It is, therefore, neither obvious that social distancing violations should take place, nor that increasing levels of crowding at the levels we observed should automatically give rise to increasing numbers of social distancing violations.

The reliability of the computer vision algorithm was assessed by comparing automated ratings of a subset of still frames against manual ratings by trained human coders, who had reference objects (eg, street tiles) available to evaluate the metric distance between those present. The human-computer interrater reliability assessment yielded excellent Krippendorff α scores larger than 0.8 (for further details, see Bernasco et al [14] and Krippendorff [25]). For practical reasons, this evaluation was conducted using situational counts of social distancing violations per still frame rather than individual-level records, which would have been preferred given that individuals are used as the unit of analysis in some of the current analyses. However, given the excellent interrater reliability score, we here assume that the corresponding individual-level score for social distancing would be at least acceptable. All statistical analyses were run in Stata 16 (StataCorp) and R (The R Foundation).

### Ethical Considerations

Informed consent was neither feasible to obtain nor required under prevailing ethical guidelines, which exempt researchers from obtaining consent for naturalistic observation conducted in public settings (American Psychological Association’s Ethical Principles, 2017, Section 8.03 [26]). No personally identifiable information was coded from the footage, and the resulting statistical dataset was fully anonymized. No compensation was offered. The study was approved by the Ethics Review Board of the Faculty of Social and Behavioral Sciences at the University of Amsterdam (2021-AISSR-14225).

## Results

Almost half of the persons observed (mean 0.44, SD 0.50) were involved in at least 1 social distancing violation. This may be considered a very high incident rate, given that the close encounters were measured from single moments (1 still frame) rather than across several moments as people moved throughout space. The median of persons present in the still frames was 7 (SD 7.7), ranging between 2 and 67 persons.

Estimated with a linear probability model [27] specified with cluster-corrected standard errors (ie, persons nested in still frames), we found a positive association between people crowding and social distancing violations at a very conservative α level of .0005 (B=.009, 99.95% CI 0.009-0.010). This means that for each additional 10 people present, the likelihood of a pedestrian failing to comply with social distancing increases by 9 percentage points.

The above result remained robust across a number of alternative specifications, including adjustments for recording camera (B=.011, 99.95% CI 0.010-0.011) and noncompliance measured alternatively as close encounters with 2 or more persons (B=.006, 99.95% CI 0.005-0.006). The latter operationalization was meaningful because it plausibly excluded many close encounters with household members with whom the person was walking side-by-side [28], focusing instead on close encounters with strangers (ie, social distancing measures concern close encounters with strangers). Finally, we identified extreme values in our crowding measure as those exceeding the upper outer fence (Q3+3×IQR, or values above 60). After winsorizing these extremes (0.11% of data) by capping them at 60, the above result remained unaltered (B=.009, 99.95% CI 0.009-0.010).

**Figure 1.**
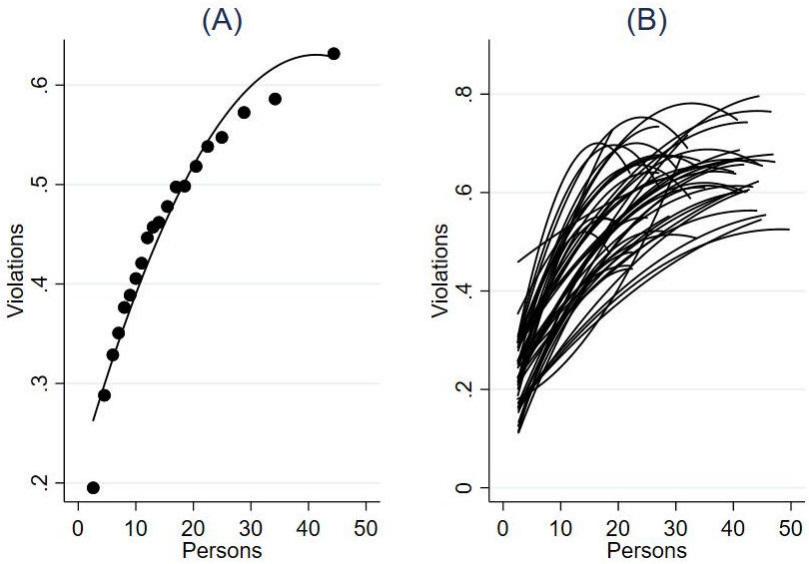
Association between people crowding and the individual-level likelihood of social distancing violations (A) across cameras and (B) per camera. Plots based on video-observational data from Amsterdam, March 2020 to June 2021 (n=539,127).

Next, to evaluate the functional form, we visualized the association with binned scatterplots of the unwinsorized raw data [29]. As shown in Figure 1A, the analysis suggests that the association is curvilinear, with an initial steep slope that decreases with larger crowd counts. In other words, the individual probability of violating social distancing recommendations increases when more people are present, but this increase stagnates as the crowd grows larger. From this graph, it may also be established that people crowding has a substantial accumulating effect on noncompliance—in a situation where there are 30 individuals present, each individual has around 60% likelihood of transgressing the social distancing recommendations.

The size and layout of the video-captured streets vary among the cameras in the sample. For example, some cameras capture streets while others capture wide squares. These variations may influence the density of people present, and thus, in turn, the likelihood of violations (eg, if people are more densely crowded in a small area, it may be more difficult to maintain distance). To account for this variation, we reran the analysis for each camera in the sample to assess whether the link between crowding and social distancing remained consistent across different cameras or settings. This visualization for each camera is presented in Figure 1B. This figure shows that although there is some variation between the cameras, the relationship between crowding and individual violation propensity was consistently positive and stagnant as group size increased across the 49 cameras in the sample.

It should be acknowledged that the above analysis plausibly overestimates the association due to spatial autocorrelation [30]. With 2 persons in a still frame, their potential violation acts are not just interdependent but are inherently linked since the physical distance between the 2 individuals is always the same for both. One solution is to use the still frames, rather than individuals, as the unit of analysis, with the outcome measured alternatively as an aggregate count of individual violations per still frame.

**Figure 2.**
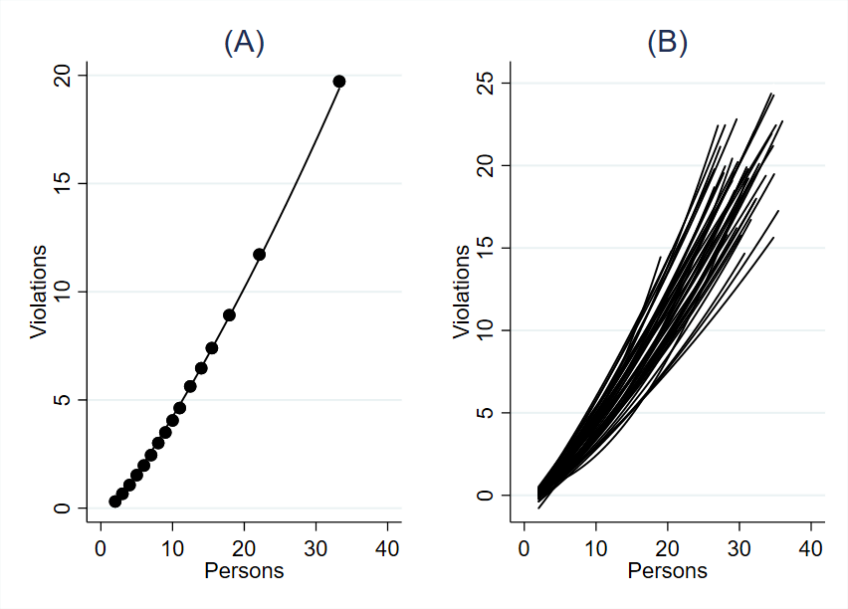
Association between people crowding and the situational-level frequency of social distancing violations. (A) Across cameras. (B) Per camera. Plots based on video-observational data from Amsterdam, March 2020 to June 2021 (n=56,429).

As we see in Figure 2A, it is noteworthy that the functional form was nearly linear with a slight tendency to increase curvilinearly—rather than decreasing curvilinearly at peak crowdedness (as in Figure 1). Regressing the number of violations on the number of persons present resulted in an unstandardized coefficient of 0.61 (99.5% CI 0.61-0.62), suggesting approximately 6 additional violations for every 10 additional persons present, with a very large *R*² of 0.80. The frequency of violations in a still frame thus grows at a steady positive rate as the number of people present increases. Although the individual and situational analyses suggest different functional forms, both indicated that the association was sizeable and positive in nature.

In Figure 2B, we again estimate the effect of people crowding on the number of social distancing violations within each of the cameras in the sample to see if the heterogeneity of the cameras might influence this relationship. While there was some variation between the cameras in the sample, the graph showed a consistent positive relationship between people crowding and the number of social distancing violations for each of the cameras.

One might argue that the increased risk of social distancing violations and crowding is trivial, as more people inherently raise the risk of close contact. To determine whether the findings are merely a result of this logical deduction or if a behavioral component is also at play, we simulated this logical baseline and compared it to our empirical results. To establish this baseline, we positioned fixed numbers of individuals randomly in a fictive rectangle the size of the average camera viewshed (650 m²), and counted the number of pairs that were within 1.5 meters distance of each other. This setup of random location assignment assumes that when positioning themselves in space, individuals are neither attracted nor repelled by others, and thus, do not have a specific preference for distance from or proximity to others. To account for the possibility that not all 650 m² in the camera viewsheds could be easily occupied by pedestrians, we repeated the simulation with reduced areas of 300 and 150 m².

**Figure 3.**
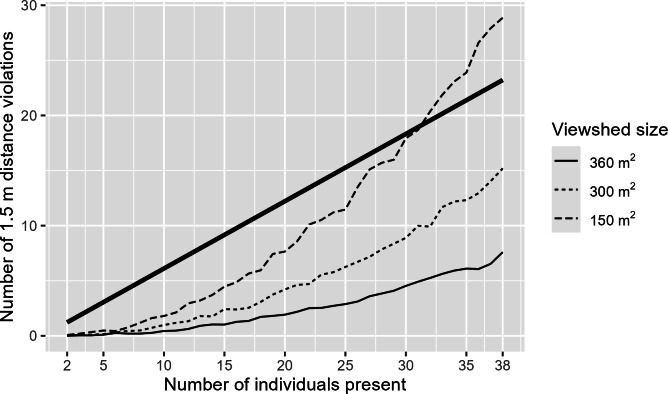
Observed (bold line) and simulated (3 thin lines) association between people crowding and frequency of social distancing violations.

Figure 3 illustrates that under this design of random location assignment, the number of violations increases exponentially with the number of people present. It also shows that, over most of the observed range of crowding, the number of violations (bold straight line) is substantially larger than the number expected if navigational choices were random. The areas of the street should thus, in principle, offer enough space for it to be possible for people to be copresent without being too close. However, people seem to prefer to stay within 1.5 meters of others rather than keep a larger distance—a tendency that reverses only in very high-density settings, with large crowds concentrated in spaces smaller than the real-life average (such crowding levels are very rare in our data: levels above 25 persons occur in less than 5 percent of our still frames, and levels above 38 in less than 1 percent).

Our data does not shed light on the behavioral reason for this nonobvious tendency. However, one explanation could be that people have a tendency to gravitate toward the center of the street, walking in each other’s footsteps and proximity. This could mirror cultural norms for appropriate pedestrian behavior [31] or suggest that the midstreet offers pedestrians an optimal visual overview, which is a critical determinant for pedestrian movement flows [32]. Additionally, the tendency for proximity can likely be attributed to people being with their household members [28], who were exempt from social distancing directives.

## Discussion

### Principal Findings

Using an automated computer vision annotation algorithm, we found robust evidence for a positive association between people crowding and social distancing noncompliance. This result is in line with empirical studies on the link between interpersonal distance and crowding conducted in COVID-19-related [14,16] and other public settings [20].

Our results challenge the common focus on individual factors (like age, gender, and motivation) as primary explanations for distancing noncompliance. Instead, we highlight the importance of situational factors—thus addressing a call for research on situational, rather than individual-level, explanations of social distancing compliance [4]. Specifically, this study demonstrates that distancing noncompliance is influenced by the “situational opportunity” created by crowding [19]. The effect of this crowding rapidly accumulates to a magnitude that leaves little room for individual-level influences [33]. In other words, if you are in a crowded setting, chances are high that you will be involved in close encounters regardless of who you are and your willingness to distance. The situational opportunity perspective was originally developed within the field of criminology as an alternative to individual-centered explanations of crime [19], and our argument adds credence to the view that this theory generalizes to COVID-19-related rule violations (see also Liu et al [5]).

The findings of this study hold the potential to assist epidemiologists in incorporating real-life behavior into predictive models of airborne contagious diseases like COVID-19. The strong correlation between crowding and social distancing violations suggests that crowding may serve as a valid proxy measure for distancing behavior. Further, the number of people present is a straightforward measure that can easily be automated with computer vision across surveillance cameras. This is thus a flexible, low-cost, and scalable measure that epidemiologists can incorporate into prediction models of contagious diseases like COVID-19. While more advanced technology can estimate the distance between people in video footage, this study shows that simply counting the number of people present is a sufficient and reliable proxy measure of social distancing violations during the COVID-19 pandemic. Future research should explore whether this behavioral measure can help predict disease incidence effectively.

Our results also have potential practical implications for how policy makers may increase public adherence to social distancing measures. Public health information has centered around keeping interpersonal distance, while the other aspect of social distancing—to avoid gatherings and crowded places—has arguably received less attention. Our findings question that focus, given that social distancing violations are a direct function of crowding. Thus, at least in urban public settings, the safest way to optimize compliance seems to be avoidance of crowded places altogether, and such a recommendation could counter the misleading idea that one may maneuver through a crowded place while keeping interpersonal distance.

This argument could be taken one step further by simply recommending that policy makers focus less on individually targeted interventions—such as information campaigns—and more on situational interventions, such as crowd management strategies [34]. In tailoring such crowd management, the emphasis may not be on creating more street space for social distancing. Our analysis indicates that existing streets already, in principle, offer enough room for copresent pedestrians to maintain the requested 1.5-meter distance (see Figure 3). In this view, the challenge seems to lie, in people’s tendency to gravitate toward other people when moving through public space. Therefore, successful interventions aimed at increasing distance compliance could consider this crowd dynamic, instead of expanding physical space.

### Limitations

It should be acknowledged as a limitation of the current analysis that it did not include any individual-level predictors—either because it was beyond the technological capacity of the computer vision tool developed to detect these properties (eg, age) or because it would have required us to survey the observed persons (eg, risk perceptions), which was not practically feasible. Therefore, we cannot directly compare the effect size of these against the situational effect of people crowding and examine how they may interact. We also did not measure whether individuals wore face masks or other kinds of protective equipment, which could potentially influence the likelihood of people complying with social distancing recommendations. For instance, the risk compensation theory posits that wearing masks might give a false sense of security, potentially causing people to relax their distancing efforts. However, it is important to note that this hypothesis lacks consistent behavioral evidence [17,35]. Further, we acknowledge that our results may not generalize to nonurban rural areas or private, semipublic, and indoor settings. For example, patterns of human presence in indoor places tend to be more socially programmed than outdoors [36], indicating that the crowding-encounter link may be less mechanically direct indoors vis-a-vis outdoors. More broadly, it is important to consider that interpersonal distance is shaped by cultural norms [37], highlighting potential constraints on the applicability of our findings across diverse cultural contexts.

Finally, it should be stressed that our analysis focuses on behavioral compliance without assuming that noncompliance with distancing leads to interpersonal virus transmission. In fact, the high prevalence of violations does not necessarily pose an epidemiological problem, given that the observed encounters are mostly brief, nonverbal, and occur outdoors, thus implying a relatively low risk for coronavirus transmission [38]. While this study introduces a readily available measure to study the spread of airborne contagious diseases, the intention of this paper is not to argue that our measure alone suffices to predict transmission rates. Depending on the disease under scrutiny, a number of other mitigating and aggravating variables should be accounted for in addition to the behavioral measures developed in this paper.

## References

[1] Haug N, Geyrhofer L, Londei A (2020). Ranking the effectiveness of worldwide COVID-19 government interventions. Nat Hum Behav.

[2] Hevey D, Perk J, Gohlke H, Hellemans I, Sellier P, Mathes P, Monpère C (2007). Cardiovascular Prevention and Rehabilitation.

[3] Davies R, Mowbray F, Martin AF, Smith LE, Rubin GJ (2022). A systematic review of observational methods used to quantify personal protective behaviours among members of the public during the COVID-19 pandemic, and the concordance between observational and self-report measures in infectious disease health protection. BMC Public Health.

[4] Kooistra EB, van Rooij B (2020). Pandemic compliance: a systematic review of influences on social distancing behaviour during the first wave of the COVID-19 outbreak. SSRN Journal.

[5] Liu N, Folmer CPR, Lo CH, Rooij B (2023). Situational voluntary compliance. China Rev.

[6] van Rooij B, de Bruijn AL, Folmer CR (2020). Compliance with COVID-19 mitigation measures in the United States. SSRN J.

[7] Hansen PG, Larsen EG, Gundersen CD (2022). Reporting on one’s behavior: a survey experiment on the nonvalidity of self-reported COVID-19 hygiene-relevant routine behaviors. Behav Public Policy.

[8] Jerolmack C, Khan S (2014). Talk is cheap: ethnography and the attitudinal fallacy. Sociol Methods Res.

[9] Koher A, Jørgensen F, Petersen MB, Lehmann S (2021). Monitoring public behavior during a pandemic using surveys: proof-of-concept via epidemic modelling. https://raw.githubusercontent.com/mariefly/HOPE/master/Monitoring_Public_Behavior_During_a_Pandemic_Using_Surveys_Proof-of-concept_Via_Epidemic_Modelling_20210831.pdf.

[10] Deutscher I, Pestello FP, Pestello HFG (1993). Sentiments and Acts.

[11] Gilmore RO, Adolph KE (2017). Video can make behavioural science more reproducible. Nat Hum Behav.

[12] Nassauer A, Legewie NM (2021). Video data analysis: a methodological frame for a novel research trend. Sociol Methods Res.

[13] Benton JS, French DP (2024). Untapped potential of unobtrusive observation for studying health behaviors. JMIR Public Health Surveill.

[14] Bernasco W, M. Hoeben E, Koelma D (2023). Promise into practice: application of computer vision in empirical research on social distancing. Sociol Methods Res.

[15] Goldstein Y, Legewie NM, Shiffer-Sebba D (2023). 3D social research: analysis of social interaction using computer vision. Sociol Methods Res.

[16] Hoeben EM, Bernasco W, Liebst LS, van Baak C, Lindegaard MR (2021). Social distancing compliance: a video observational analysis. PLOS One.

[17] Liebst LS, Ejbye-Ernst P, de Bruin M, Thomas J, Lindegaard MR (2022). No evidence that mask-wearing in public places elicits risk compensation behavior during the COVID-19 pandemic. Sci Rep.

[18] Munafò MR, Nosek BA, Bishop DVM (2017). A manifesto for reproducible science. Nat Hum Behav.

[19] Wilcox P, Cullen FT (2018). Situational opportunity theories of crime. Annu Rev Criminol.

[20] Kaya N, Erkíp F (1999). Invasion of personal space under the condition of short-term crowding: a case study on an automatic teller machine. J Environ Psychol.

[21] Latané B (1981). The psychology of social impact. Am Psychol.

[22] Fischer P, Krueger JI, Greitemeyer T (2011). The bystander-effect: a meta-analytic review on bystander intervention in dangerous and non-dangerous emergencies. Psychol Bull.

[23] Philpot R, Liebst LS, Levine M, Bernasco W, Lindegaard MR (2020). Would I be helped? Cross-national CCTV footage shows that intervention is the norm in public conflicts. Am Psychol.

[24] Hasan I, Liao S, Li J, Akram SU, Shao L Generalizable pedestrian detection: the elephant in the room.

[25] Krippendorff K (2004). Reliability in content analysis: some common misconceptions and recommendations. Hum Commun Res.

[26] (2017). Ethical principles of psychologists and code of conduct. American Psychological Association.

[27] Breen R, Karlson KB, Holm A (2018). Interpreting and understanding logits, probits, and other nonlinear probability models. Annu Rev Sociol.

[28] Liebst LS, Baggesen L, Dausel KL, Pallante V, Lindegaard MR (2023). Human observers are accurate in judging personal relationships in real-life settings: a methodological tool for human observational research. Field Methods.

[29] Starr E, Goldfarb B (2020). Binned scatterplots: a simple tool to make research easier and better. Strategic Manage J.

[30] Ward MD, Gleditsch KS (2018). Spatial Regression Models.

[31] Jensen OB (2010). Negotiation in motion: unpacking a geography of mobility. Space Cult.

[32] Turner A, Penn A Making isovists syntactic: isovist integration analysis. https://www.researchgate.net/profile/Alan-Penn-2/publication/242075155_Making_isovists_syntactic_isovist_integration_analysis/links/0046352bbef92ce31e000000/Making-isovists-syntactic-isovist-integration-analysis.pdf.

[33] Funder DC, Ozer DJ (2019). Evaluating effect size in psychological research: sense and nonsense. Adv Methods Pract Psychol Sci.

[34] Johansson A, Batty M, Hayashi K, Al Bar O, Marcozzi D, Memish ZA (2012). Crowd and environmental management during mass gatherings. Lancet Infect Dis.

[35] Mantzari E, Rubin GJ, Marteau TM (2020). Is risk compensation threatening public health in the covid-19 pandemic?. BMJ.

[36] Hanson J (2003). Decoding Homes and Houses.

[37] Hall ET (1966). The Hidden Dimension.

[38] Bulfone TC, Malekinejad M, Rutherford GW, Razani N (2021). Outdoor transmission of SARS-CoV-2 and other respiratory viruses: a systematic review. J Infect Dis.

[39] For review: social distancing violations as a situational correlate of crowding. OSF.

